# 1438. Hand Hygiene Compliance and Monitoring among Healthcare Workers at an Urban Hospital in the Caribbean.

**DOI:** 10.1093/ofid/ofad500.1275

**Published:** 2023-11-27

**Authors:** Ezinne U M Nwaogu

**Affiliations:** Doctors Hospital, Nassau, New Providence, The Bahamas

## Abstract

**Background:**

Hand hygiene is one of the most effective ways to prevent the spread of infections in the Hospital.

To promote quality care, simple procedures like hand hygiene are of paramount importance.

To prioritize clean hands in health facilities, people at all levels need to believe in the importance of hand hygiene by acting as key players in achieving appropriate behaviors and attitudes towards it.

**Methods:**

A cross-sectional observational study was used, by direct observation technique. 'Secret shoppers' employees, were used to monitor colleagues' hand hygiene compliance each time they enter or exit a patient's room.

Role modeling, make it part of our rounding/just-in-time education, infection prevention, and control (IPC) monthly hand hygiene compliance updates/feedback, and incentives. We mention it in every huddle, especially Healthcare-Associated Infections (HAI) meetings, Electronic platforms, mandatory training and education for all Employees.

**Results:**

The findings of this study showed that our hand hygiene compliance rates increased from 52% to 87% across most of the units - ICU, IMCU, Medical Surgical unit, Maternity, Emergency Room, OR and by all employees ranging from physicians to nurses, patient care technicians and others.

Healthcare workers, now aware they are being monitored, by secret shoppers, work in competition with other units to improve their compliance because they would be given the recognition " hand hygiene champions. "

**Conclusion:**

In conclusion, using secret shoppers and positive recognition helped our Hospital to achieve a higher degree of compliance with hand hygiene.

More will still need to be done, to get our compliance closer to 100%

Clean Hands Save Lives! Together, we can achieve it.

**Disclosures:**

**All Authors**: No reported disclosures

